# G378X‐I148T CFTR variant: A new complex allele in a cystic fibrosis newborn with pancreatic insufficiency

**DOI:** 10.1002/mgg3.2033

**Published:** 2022-08-13

**Authors:** Vito Terlizzi, Claudia Centrone, Matteo Botti, Giovanni Taccetti

**Affiliations:** ^1^ Department of Paediatric Medicine Cystic Fibrosis Regional Reference Center Meyer Children's Hospital Florence Italy; ^2^ Diagnostic Genetics Unit Careggi University Hospital Florence Italy; ^3^ Tuscany Support Cystic Fibrosis Service, Department of Pediatrics Leghorn Hospital Leghorn Italy

Dear Editor,

Cystic Fibrosis (CF) is a multisystem disease caused by mutations causing deficient or dysfunctional CF transmembrane conductance regulator (*CFTR*) protein. Today, the growing proportion of newborn screening (NBS) programmes and the use of *CFTR* gene sequencing lead frequently to the detection of rare variants with no clear genotype–phenotype correlation or the identification of complex alleles. This complicates genetic counselling. They result from the combination of two or more *CFTR* variants in cis (i.e. on the same allele) that sometimes can act as a pathogenic variant, whereas each single variant has only a minor or no effect (Terlizzi et al., 2017).

Here, we report the case of a Caucasian newborn resulted positive for CF NBS, according to the algorithm blood immunoreactive trypsinogen (b‐IRT)‐meconium lactase‐DNA‐sweat chloride of CF centre of Florence, Italy (Terlizzi et al., 2020). The informed consent was obtained from the parents of the infant after a complete description of the aims of the study.

b‐IRT was above the 99th centile (75 ng/ml vs. 50 ng/ml) with positive meconium lactase. No *CFTR* variants were identified after the first level genetic analysis, including all CF‐causing variants (https://cftr2.org/). The baby was born at 39 weeks of pregnancy with a weight of 2250 g. Soon after, she was hospitalised for poor nutritional status and steatorrhoea. Faecal elastase 1 confirmed the suspect of pancreatic insufficiency (<15 μg/g), and pancreatic enzyme replacement therapy was started, along with fat‐soluble vitamins, saline solution and respiratory physiotherapy, with a rapid weight gain. Chest x‐ray and abdominal ultrasound were normal. The sweat test confirmed the diagnosis of CF (chloride 109–110 mEq/L), and *CFTR* gene sequencing (detection rate 98%) revealed two *CFTR* variants: L867X (c.26007>A; p.Leu867X) inherited from the mother and G378X (c.1132G>T; p.Gly378Ter)‐I148T in complex allele, inherited from the father. Now, the infant has 12 months, and we are continuing the follow‐up, according to CF standard of care. During follow‐up, throat swab was positive for *Pseudomonas aeruginosa* at 6 and 11 months.

I148T (c.443T>C; p.Ile148Thr) is a missense *CFTR* non causing variant, based on evaluation of clinical characteristics of 114 patients carrying this variant. The determination of non‐CF‐causing variant does not exclude the possibility that this variant may contribute to CF‐like symptoms in certain individuals. In some cases, patients with I148T (combined with a CF‐causing variant) may develop mild symptoms in select organ systems and/or be diagnosed as having a CFTR‐related disorder (https://cftr2.org/). Furthermore, functional studies revealed normal processing, gating and conductance of the *CFTR* (Seibert et al., 1997). However, it was present at >100‐fold frequency in the general population compared with CF patients and is itself not associated with CF (Monaghan et al., 2004; Watson et al., 2004). Further studies revealed a second variant 3199del6, in cis with I148T in affected patients (Rohlfs et al., 2002; Ruchon et al., 2003). The 3199del6 variant was not present in asymptomatic compound heterozygous individuals with I148T and F508del (Monaghan et al., 2004). Subsequent studies identified 3199del6 in 0.9% of I148T carriers and determined the frequency of 3199del6 in the general population to be <0.1% (Buller et al., 2004). Finally, the complex allele I148T‐3199del6 caused CF in five compounds heterozygous with a class I–II variant, and their *CFTR* activity on nasal epithelial cells was comparable with patients with two class I–II variants (mean 7.3% vs. 6.9%; Terlizzi et al., 2017). Anyway, 3199del6 also occurs in the absence of I148T, as a patient with severe CF has been reported with 3199del6 (negative for I148T) and G542X (Buyse et al., 2003).

L867X *CFTR* variant is reported only in one CF Scottish adult patient carrying F508del on the other allele (http://www.genet.sickkids.on.ca/). No other information is available. G378X is not found in CFTR2 (https://cftr2.org/) and CFTR1 database (http://www.genet.sickkids.on.ca/). We report the first case of a CF newborn with pancreatic insufficiency, diagnosed for positive NBS, carrying the new complex allele I148T‐G378X and new *CFTR* variant G378X, identified after gene sequencing. We might speculate that G378X should be considered whenever I148T is identified.

## AUTHOR CONTRIBUTIONS

Vito Terlizzi and Giovanni Taccetti follow the infant, contributed to the conception, design of the paper and wrote it; Claudia Centrone performed the *CFTR* genetic analysis of the infant; Matteo Botti follows the infant. All authors read, reviewed and approved the final version of the manuscript, to be published and agreed to be accountable for all aspects of the work in ensuring that questions related to the accuracy or integrity of any part of the work are appropriately investigated and resolved.

## CONFLICT OF INTEREST

The authors declare no conflict of interest.
